# Effect of an MMA-Containing Adhesive Primer on the Surface Characteristics of Sandblasted PEEK and Its Bond Strength to Dental Resin Cements

**DOI:** 10.3390/ma19173774

**Published:** 2026-09-04

**Authors:** Taro Mukaibo, Takafumi Watanabe, Hiromichi Ogusu, Kanna Saimoto, Shoshi Ikemoto, Chihiro Masaki, Hiroshi Ikeda

**Affiliations:** 1 Division of Oral Reconstruction and Rehabilitation, Kyushu Dental University, Kitakyushu 803-8580, Japan; r07mukaibou@fa.kyu-dent.ac.jp (T.M.);; 2 Division of Biomaterials, Kyushu Dental University, Kitakyushu 803-8580, Japanr16ikeda@fa.kyu-dent.ac.jp (H.I.); 3 Division of Occlusion and Maxillofacial Reconstruction, Kyushu Dental University, Kitakyushu 803-8580, Japan

**Keywords:** poly(ether ether ketone), adhesive primer, methyl methacrylate, resin cement, shear bond strength, thermal aging

## Abstract

This study evaluated the effects of the methyl methacrylate (MMA)-containing adhesive primer Visio.link on the surface characteristics of sandblasted poly(ether ether ketone) (PEEK) and its bond strength to four dental resin cements: the MMA-based resin cement Super-Bond EX (SB) and the composite-based resin cements RelyX Universal Resin Cement (RX), G-CEM LinkForce (GC), and Panavia V5 (PA). Sandblasted PEEK specimens were tested with or without primer application. Surface morphology, surface roughness, and surface free energy were evaluated, and shear bond strength (SBS) was measured after 1 day of water storage and after 20,000 thermal cycles. Primer application smoothed the sandblasted surface, reduced surface roughness, increased the polar component, and decreased the dispersive component and total surface free energy. Primer application significantly increased the initial SBS of RX, GC, and PA; however, thermal aging reduced bonding durability and caused pretest debonding in several groups. For SB, primer application did not significantly affect the initial SBS, whereas the sandblasted group showed significantly higher SBS than the primer-treated group after aging. The effect of the MMA-containing primer therefore depended on resin cement type and aging condition. These findings suggest that primer application should be considered according to the composition and bonding mechanism of the resin cement rather than routinely incorporated into PEEK bonding procedures.

## 1. Introduction

Poly(ether ether ketone) (PEEK) is a high-performance thermoplastic polymer with favorable mechanical properties, physicochemical stability, and biocompatibility [1]. Owing to these characteristics, PEEK has been widely used in industrial, medical, and dental applications [2,3]. In dentistry, PEEK has been used for implant superstructures, abutments, removable and fixed prosthetic frameworks, denture bases, crowns, and post-and-core restorations [4,5]. Several clinical studies have also investigated the performance of PEEK-based prosthetic devices [6,7,8,9,10]. Despite these advantages, PEEK has an intrinsically opaque appearance and limited translucency, resulting in inferior esthetic properties compared with ceramic and resin-based restorative materials. Therefore, PEEK frameworks are often veneered with esthetic resin-based materials [11,12,13]. In addition, when PEEK is used for crowns or fixed dental prostheses, reliable bonding to luting agents is essential for clinical success. However, PEEK is chemically inert and has low surface energy, resulting in poor adhesion to conventional dental adhesives and functional monomers, including silane coupling agents. Inadequate bonding may lead to debonding and failure of PEEK-based prostheses. Therefore, improving adhesion to PEEK is essential for the long-term clinical performance of PEEK restorations.

Various surface treatments have been investigated to improve bonding to PEEK by increasing surface roughness and bonding area, enhancing micromechanical interlocking, or chemically modifying the surface [14]. Reported methods include chemical etching with concentrated sulfuric acid [15,16], alumina airborne-particle abrasion [17,18], tribochemical silica coating [19,20], laser irradiation [21,22], and plasma treatment [23,24]. Among these methods, etching with concentrated sulfuric acid and alumina airborne-particle abrasion have been shown to effectively improve bond strength to PEEK [25,26]. However, the clinical use of concentrated sulfuric acid is limited by its high corrosiveness and associated safety concerns, particularly for chairside procedures. In contrast, alumina airborne-particle abrasion is relatively simple and clinically applicable, making it a practical pretreatment method for bonding to PEEK.

In addition to surface pretreatment, the selection of adhesive primers and resin cements is critical for achieving durable bonding to PEEK. Previous studies have demonstrated favorable bonding of MMA-based materials to PEEK [27,28,29,30,31]. For example, Visio.link, an MMA-containing adhesive primer, has been reported to enhance the bond strength between PEEK and resin-based veneering materials [28,29], while MMA-based resin cements, such as Super-Bond, have shown more favorable bonding performance to PEEK than conventional composite-based resin cements [30,31]. In contrast, conventional composite-based resin cements generally show limited direct bonding to PEEK, likely because of the low chemical reactivity and low surface energy of the PEEK surface. An MMA-containing adhesive primer may therefore serve as an intermediate resin layer between sandblasted PEEK and composite-based resin cements, potentially improving their bonding performance. However, it remains unclear whether such a primer can compensate for the limited intrinsic bonding capability of composite-based resin cements to PEEK and whether any improvement can be maintained after aging.

Therefore, the objective of this in vitro study was to evaluate the effects of an MMA-containing adhesive primer on the surface characteristics of sandblasted PEEK and its bond strength to different resin cements, including an MMA-based resin cement and composite-based resin cements, before and after thermal aging. The following null hypotheses were tested: (1) application of an MMA-containing adhesive primer would not affect the surface morphology, surface roughness, or surface free energy of sandblasted PEEK; and (2) application of an MMA-containing adhesive primer would not affect the shear bond strength between sandblasted PEEK and resin cements, regardless of resin cement type or aging condition.

## 2. Materials and Methods

### 2.1. Materials

Table 1 lists the four commercially available resin cements used in this study: one MMA-based resin cement, Super-Bond EX (SB), and three composite-based resin cements, RelyX Universal Resin Cement (RX), G-CEM LinkForce (GC), and Panavia V5 (PA). Visio.link (bredent GmbH & Co. KG, Senden, Germany), an MMA-containing adhesive primer, was also used. A commercially available CAD/CAM PEEK block (SHOFU PEEK, Shofu, Kyoto, Japan), measuring 14.5 mm × 12.0 mm × 18.0 mm, was used as the adherend material.

The PEEK block was sectioned into rectangular specimens (14.5 mm × 12.0 mm × 2.0 mm; thickness tolerance, ± 0.1 mm) using a diamond wheel saw (SCAN-DIA GmbH & Co. KG, Hagen, Germany). The bonding surfaces were polished with 600-grit silicon carbide paper (WTCC-S, Mipox, Tochigi, Japan) and ultrasonically cleaned in distilled water for 5 min. The specimens were then subjected to alumina airborne-particle (050A, Akiyama-sangyo, Osaka, Japan) abrasion using 50 μm alumina particles at 0.2 MPa for 10 s, with the nozzle positioned 10 mm from and perpendicular (90°) to the PEEK surface. After abrasion, residual alumina particles were removed from the surfaces using an air blower (SP-AIR100, Surprise, Osaka, Japan). 

The sandblasted PEEK specimens were divided into two surface-condition groups: sandblasted PEEK without primer application (sandblasted group) and sandblasted PEEK treated with Visio.link (primer-treated group). In the primer-treated group, a single thin layer of Visio.link was applied once with a microbrush to cover the entire sandblasted PEEK bonding surface. The amount of primer was not volumetrically measured. No additional air-drying step was performed. The primer was then immediately light-polymerized for 90 s using an α-Light II light-curing unit (J. Morita Corp., Kyoto, Japan), with an emission wavelength range of 400–600 nm. The specimens from both groups were then used for the subsequent experiments.

### 2.2. Scanning Electron Microscopy

**Figure 1:**
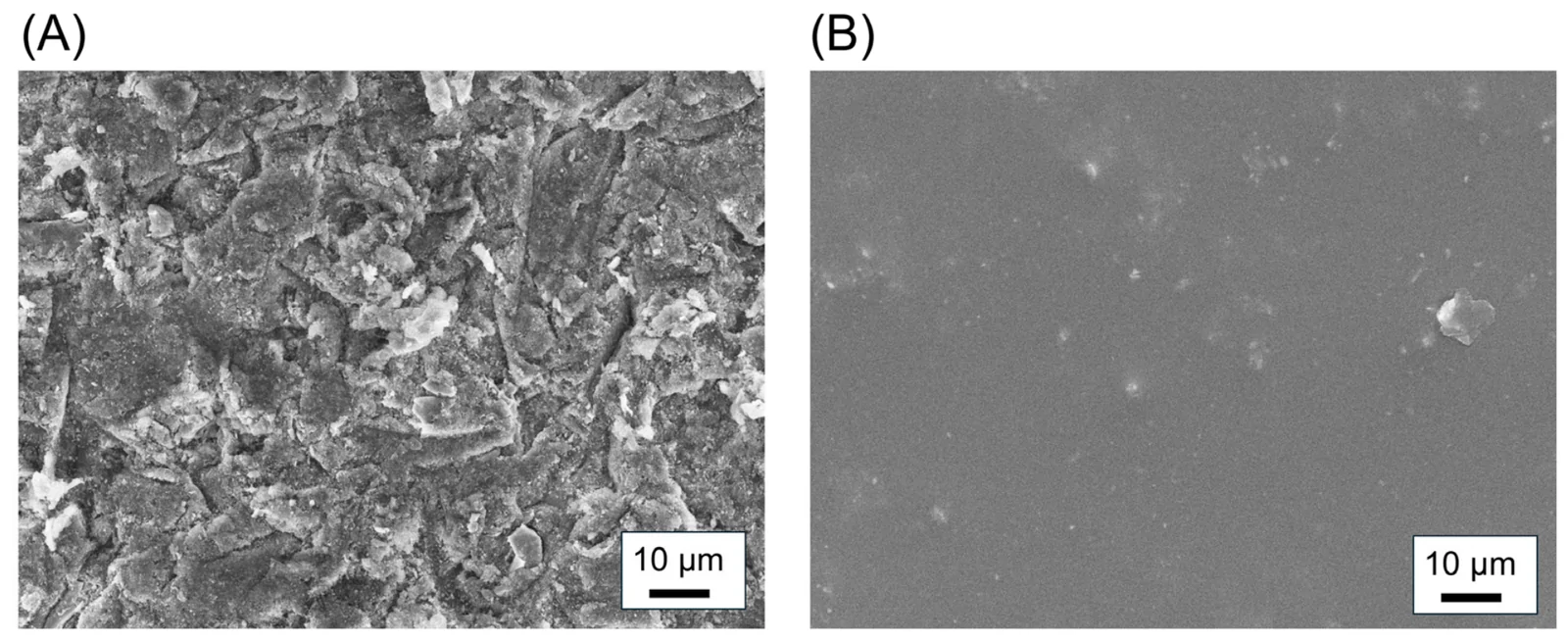
Representative SEM images of (**A**) sandblasted PEEK and (**B**) primer-treated sandblasted PEEK. Original magnification: ×1000; scale bar = 10 μm.

The surface topography of the PEEK specimens in each group was examined using scanning electron microscopy (SEM). Three independently prepared specimens from each surface-condition group were examined. Before observation, the specimen surfaces were sputter-coated with a thin layer of gold. SEM images were obtained using a JCM-7000 microscope (JEOL, Tokyo, Japan) at an accelerating voltage of 15 kV and a magnification of ×1000. Multiple areas of each specimen were observed, and the images presented in Figure 1 were selected as representative images showing the typical surface morphology consistently observed among the examined specimens.

### 2.3. Surface Roughness Measurement

The surface roughness of the PEEK specimens in each group was measured using a stylus profilometer (HANDYSURF+, Tokyo Seimitsu Co., Ltd., Tokyo, Japan). The arithmetic mean roughness (Ra) was determined at the center of each specimen over a tracing length of 10 mm. Ten independent specimens were evaluated per group, with one measurement obtained from each specimen (n = 10).

### 2.4. Surface Free Energy

The surface free energy (SFE) of the PEEK specimens in each group was determined from contact angle measurements. The contact angles of two test liquids, distilled water and diiodomethane (>99%, Kanto Chemical Co., Inc., Tokyo, Japan), were measured using a contact angle meter (DMe-211, Kyowa Interface Science Co., Ltd., Saitama, Japan) under ambient conditions at room temperature (20 ± 3 °C). For each measurement, a 2 μL droplet of the test liquid was placed on the specimen surface, and the contact angle was recorded 5 s after droplet deposition. The measurement location was selected as close as possible to the center of each specimen surface. For all specimens, the test liquids were applied in a standardized order, with distilled water measured first, followed by diiodomethane. Ten independent specimens were evaluated per group, and ten measurements were performed for each test liquid on each specimen. The mean contact angle for each specimen was used for the SFE calculation (n = 10).

The SFE was calculated from the measured contact angles using analytical software (FAMAS ver.5.0.20, Kyowa Interface Science Co., Ltd.) according to the Owens–Wendt method [32], as expressed by the following equations:

(1) $$\sqrt{{\;\gamma_{L1}^{d}\;\gamma }_{s}^{d}}+\sqrt{{\;\gamma_{L1}^{p}\;\gamma }_{s}^{p}}=\frac{\gamma_{L1}^{total}\left(1+\cos\theta_{L1}\right)}{2}$$

(2) $$\sqrt{{\;\gamma_{L2}^{d}\;\gamma }_{s}^{d}}+\sqrt{{\;\gamma_{L2}^{p}\;\gamma }_{s}^{p}}=\frac{\gamma_{L2}^{total}\left(1+\cos\theta_{L2}\right)}{2}$$

(3) $$\gamma^{total}=\gamma^{d}+\gamma^{p}$$

where the subscripts *L*1 and *L*2 denote distilled water and diiodomethane, respectively. The symbols *γ_S_^total^*, *γ_S_^p^*, and *γ_S_^d^* represent the total SFE, polar component, and dispersive component of the specimen surface, respectively. The symbols *γ_L_^total^*, *γ_L_^p^*, and *γ_L_^d^* represent the corresponding surface-tension components of the test liquids, and *θ* represents the measured contact angle. The surface-tension values used were as follows: distilled water, *γ_L_*_1_*^total^* = 72.8 mN/m, *γ_L_*_1_*^p^* = 51.0 mN/m, and *γ_L_*_1_*^d^* = 21.8 mN/m; and diiodomethane, *γ_L_*_2_*^total^* = 50.8 mN/m, *γ_L_*_2_*^p^* = 1.3 mN/m, and *γ_L_*_2_*^d^* = 49.5 mN/m.

### 2.5. Shear Bond Strength Test

The sandblasted and primer-treated PEEK specimens were used for the shear bond strength (SBS) test. Bond strength between the PEEK specimens and each resin cement was evaluated using the method described in our previous study [33]. A polytetrafluoroethylene tube with an inner diameter of 5 mm was fixed to the PEEK surface using double-sided tape to standardize the bonded area. Each resin cement (SB, RX, GC, and PA) was placed within the tube. For the composite-based resin cements RX, GC, and PA, the materials were dispensed through their respective automixing tips and polymerized in dual-cure mode. Light irradiation was performed from directly above the specimen for 10 s using a Pencure light-curing unit (J. Morita Corp., Kyoto, Japan) at an irradiance of 1000 mW/cm^2^. SB was prepared using the standard bulk-mixing procedure. Four drops of Quick Monomer (Sun Medical, Moriyama, Japan) were mixed with one drop of Catalyst V (Sun Medical, Moriyama, Japan), followed by the addition of one standard scoop of EX polymer powder (Sun Medical, Moriyama, Japan). The components were mixed until homogeneous, placed within the polytetrafluoroethylene tube, and allowed to chemically polymerize without light irradiation. After bonding, the specimens were immersed in distilled water at 37 °C for 1 day and designated as the initial group. Additional specimens were subjected to 20,000 thermal cycles between water baths maintained at 5 °C and 55 °C, with a dwell time of 60 s in each bath [34], and designated as the aged group. For each combination of surface condition, resin cement, and aging condition, ten specimens were prepared (n = 10). A formal a priori power analysis was not performed. The sample size of 10 specimens per group was selected with reference to previous in vitro PEEK bonding studies using comparable experimental designs [30,35].

The SBS test was performed for both the initial and aged groups using a universal testing machine (AGS-H; Shimadzu Corp., Kyoto, Japan). A shear load was applied to the PEEK/resin cement interface using a knife-edge-shaped jig at a crosshead speed of 1.0 mm/min until failure. The SBS was calculated by dividing the maximum load at failure by the bonded area.

After SBS testing, the fractured surfaces were examined to determine the failure mode. Failure modes were classified into four categories: adhesive failure at the PEEK/resin cement interface, cohesive failure within the resin cement, mixed failure involving both adhesive and cohesive failures, and pretest debonding. Specimens exhibiting pretest debonding before SBS testing were classified separately because the fracture interface could not be directly examined.

### 2.6. Statistical Analysis

All statistical analyses were performed using R software version 4.5.2 (R Foundation for Statistical Computing, Vienna, Austria). The normality of data distribution and homogeneity of variance were assessed using the Shapiro–Wilk test and the Brown–Forsythe modification of Levene’s test, respectively. Because normality and homogeneity of variance were satisfied, the surface roughness data were analyzed using an independent-samples Student’s *t*-test. The dispersive, polar, and total SFE components were analyzed separately using independent-samples Student’s *t*-tests. No correction for multiple comparisons was applied to the SFE analysis. Because normality and homogeneity of variance were not satisfied for several groups, the shear bond strength (SBS) data were analyzed using the Mann–Whitney U test. For the SBS analysis, four planned pairwise comparisons were specified within each resin cement: (1) sandblasted versus primer-treated specimens under the initial condition, (2) sandblasted versus primer-treated specimens after aging, (3) initial versus aged specimens in the sandblasted group, and (4) initial versus aged specimens in the primer-treated group. Where SBS data were available for both comparison groups, the resulting *p*-values were adjusted using the Holm method to control the risk of type I error. Specimens that debonded before SBS testing were recorded as pretest debonding and excluded from SBS calculations and statistical comparisons. The incidence of pretest debonding was reported separately. When all specimens in either comparison group exhibited pretest debonding, no statistical test was performed. The significance level was set at *p* < 0.05.

## 3. Results

### 3.1. Surface Characteristics

Surface changes in sandblasted PEEK following adhesive primer application were evaluated by SEM, surface roughness measurement, and SFE analysis. Figure 1 shows representative SEM images of the sandblasted and primer-treated PEEK surfaces. The sandblasted PEEK surface exhibited irregular micrometer-scale depressions and protrusions produced by alumina airborne-particle abrasion. In contrast, the primer-treated surface appeared relatively smooth, suggesting that the primer covered the sandblasted surface and partially filled the surface irregularities.

**Figure 2:**
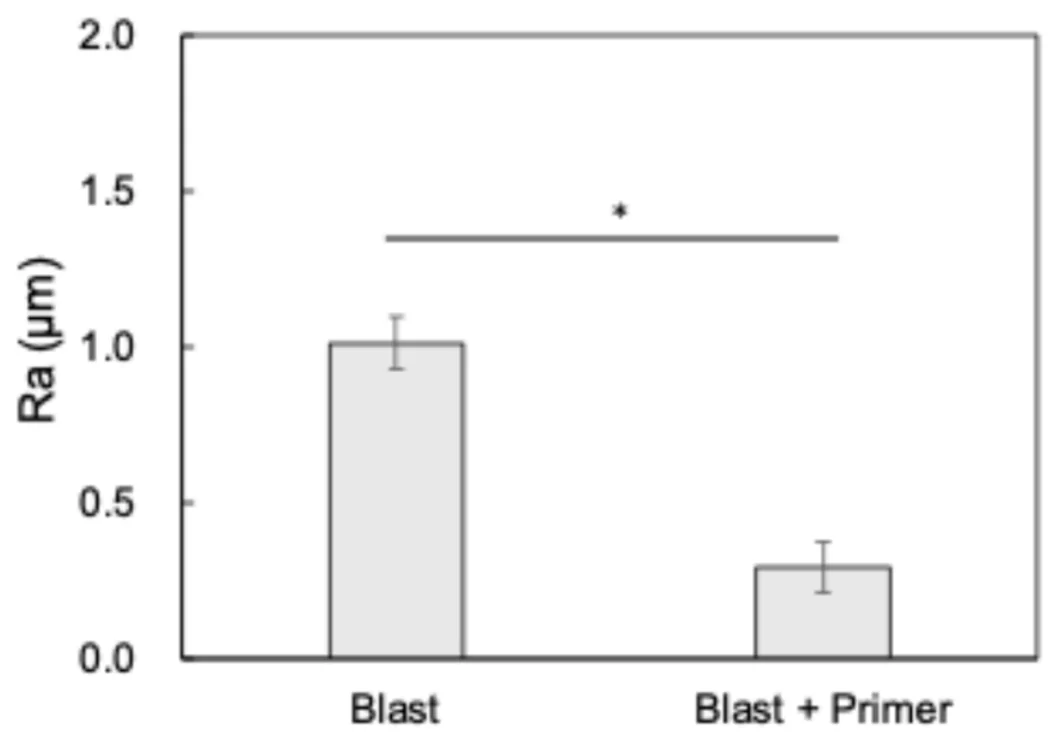
Arithmetic mean surface roughness (Ra) of the sandblasted and primer-treated PEEK surfaces. Data are presented as the mean ± standard deviation (n = 10). * *p* < 0.001.

Surface roughness was quantitatively evaluated using a stylus profilometer. Figure 2 shows the arithmetic mean roughness (Ra) of the two PEEK surfaces. The Ra value of the sandblasted surface was 1.02 ± 0.11 μm, which was significantly higher than that of the primer-treated surface (0.32 ± 0.09 μm; *p* < 0.001).

**Figure 3:**
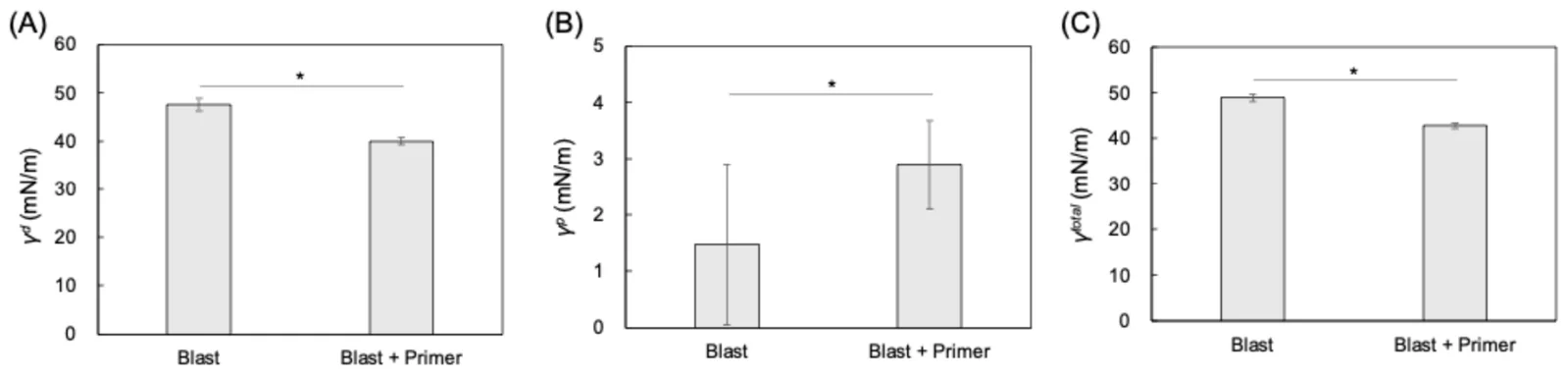
Surface free energy of the sandblasted and primer-treated PEEK surfaces: (**A**) dispersive component, (**B**) polar component, and (**C**) total surface free energy. Data are presented as the mean ± standard deviation (n = 10). * *p* < 0.001.

The SFE components of each PEEK surface are shown in Figure 3. The dispersive component was significantly higher for the sandblasted surface (47.5 ± 1.3 mN/m) than for the primer-treated surface (40.0 ± 0.7 mN/m). In contrast, the polar component was significantly lower for the sandblasted surface (1.5 ± 1.4 mN/m) than for the primer-treated surface (2.9 ± 0.8 mN/m). The total SFE was also significantly higher for the sandblasted surface (49.0 ± 0.8 mN/m) than for the primer-treated surface (42.9 ± 0.6 mN/m).

### 3.2. Shear Bond Strength and Failure Modes

**Figure 4:**
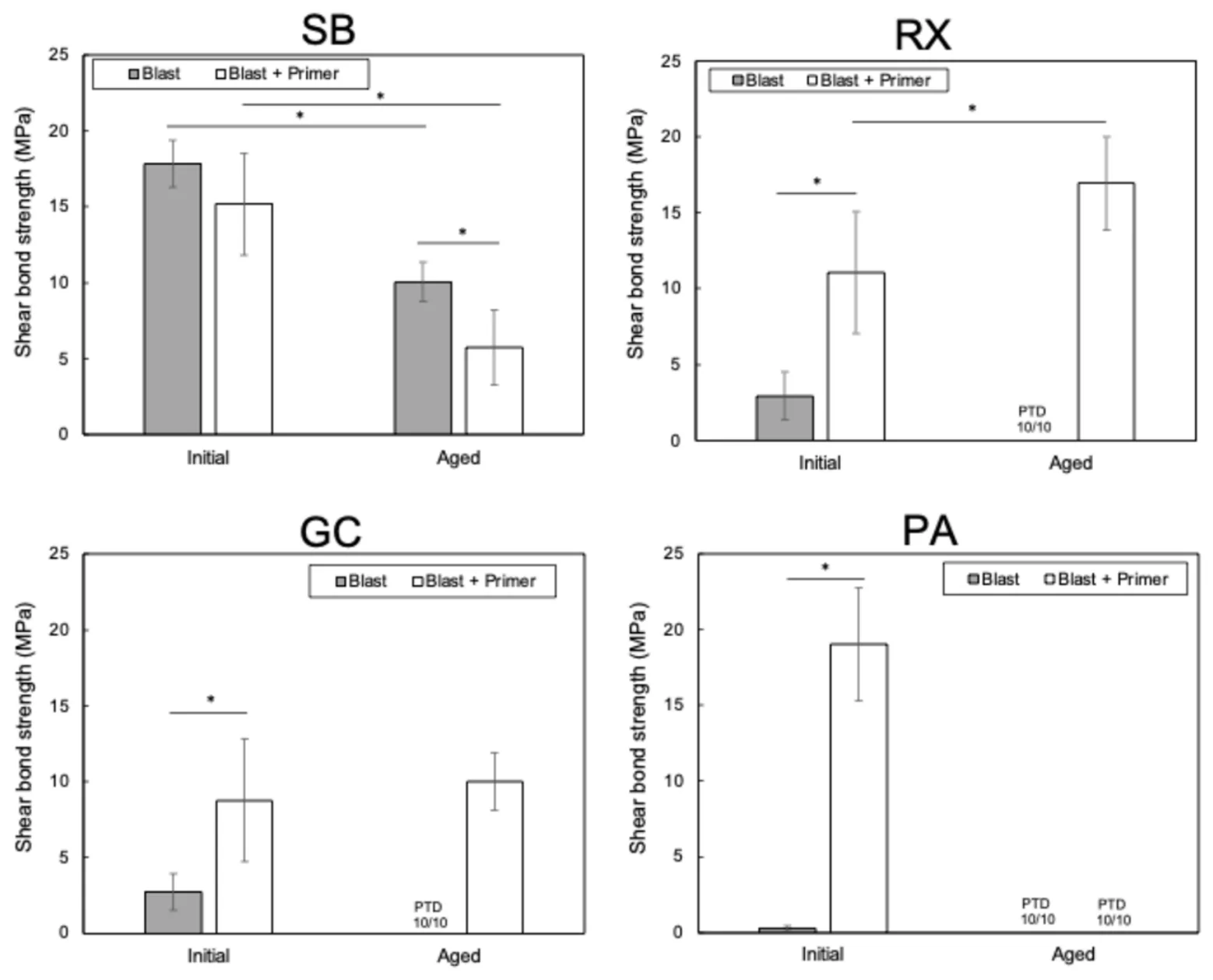
Shear bond strength of sandblasted PEEK and primer-treated PEEK bonded with each resin cement. Only specimens that successfully underwent SBS testing were included in SBS calculations. Groups in which all specimens exhibited pretest debonding are indicated as “PTD 10/10.” Data are presented as mean ± standard deviation (initial sample size: n = 10). Pretest debonding specimens were excluded from SBS calculations. * *p* < 0.01.

Figure 4 shows the SBS values for each resin cement (SB, RX, GC, and PA) under the initial and aged conditions. For SB, no significant difference in SBS was observed between the sandblasted and primer-treated groups under the initial condition. After aging, however, the SBS of the sandblasted group was significantly higher than that of the primer-treated group. For RX and GC, the initial SBS values of the sandblasted groups were significantly lower than those of the corresponding primer-treated groups. After aging, all specimens in the sandblasted RX and GC groups exhibited pretest debonding before SBS testing; therefore, SBS values could not be determined for these specimens. For PA, the initial SBS of the sandblasted group was significantly lower than that of the primer-treated group. After aging, all PA specimens in both surface-condition groups exhibited pretest debonding before SBS testing, indicating complete loss of bonding under the aging condition used in this study.

The failure modes after SBS testing and the incidence of pretest debonding are summarized in Table 2. Under the initial condition, RX, GC, and PA specimens that underwent SBS testing exhibited adhesive failure. For SB under the initial condition, two specimens in the sandblasted group exhibited mixed failure, whereas all specimens in the primer-treated group exhibited adhesive failure. After aging, all SB specimens and the successfully tested RX and GC specimens exhibited adhesive failure. Pretest debonding occurred in all specimens of the sandblasted RX and GC groups and in both PA surface-condition groups after aging and was recorded separately because the fracture interface could not be examined.

## 4. Discussion

This study investigated the effects of an MMA-containing adhesive primer on the surface characteristics of sandblasted PEEK and its bond strength to different resin cements. Primer application smoothed the PEEK surface, significantly reduced its surface roughness, and altered its surface free energy. Its effect on bond strength depended on the type of resin cement and the aging condition. Under the initial condition, primer application increased the bond strength of the composite-based resin cements RX, GC, and PA. In contrast, for the MMA-based resin cement SB, no significant effect was observed under the initial condition; however, after aging, the sandblasted group showed significantly higher bond strength than the primer-treated group. Therefore, both null hypotheses were rejected.

SEM revealed that sandblasting created irregular microscale depressions and protrusions on the PEEK surface, which may enhance micromechanical interlocking with the resin cement [27]. In contrast, the primer-treated surface appeared smoother, suggesting that the primer coated the sandblasted surface and partially filled its surface irregularities. This interpretation was supported by the significant reduction in Ra following primer application. The primer may therefore have formed an intermediate resin layer that improved interfacial compatibility with the resin cement. At the same time, however, smoothing of the abraded surface may have reduced the micromechanical retention provided by sandblasting.

Primer application also altered the surface free energy of sandblasted PEEK. The increase in the polar component suggests that the primer-treated surface exhibited greater polarity than the sandblasted PEEK surface, possibly due to the formation of a primer-derived surface layer. However, the total surface free energy decreased after primer application. These findings indicate that the bonding results cannot be explained solely by changes in surface free energy. Rather, bond strength was likely influenced by multiple factors, including wettability, surface roughness, the mechanical properties and integrity of the primer layer, the composition of the resin cement, and interactions between the primer and resin cement.

The SBS results demonstrated that the effect of primer application depended on the type of resin cement. For the composite-based resin cements RX, GC, and PA, primer application significantly increased the initial SBS. The low SBS values of these cements when applied directly to sandblasted PEEK suggest that micromechanical retention alone provides limited bonding and that these composite-based resin cements have limited intrinsic chemical interaction with PEEK. The MMA-containing primer may therefore function as an intermediate resin layer that improves interfacial compatibility between PEEK and the composite-based resin cements. These filler-containing resin cements are relatively viscous [36] and may therefore have limited ability to penetrate the microgrooves created by sandblasting. In addition, the relatively low viscosity of the MMA-containing adhesive primer may facilitate its penetration into the surface irregularities of sandblasted PEEK. Copolymerization between the primer layer and the resin cement may also have contributed to the improved initial bonding performance, although this mechanism was not directly evaluated in the present study. After aging, however, the SBS of the composite-based resin cements decreased, and some groups exhibited pretest debonding. These findings indicate that interfacial degradation occurred during thermal aging, even when primer application improved the initial SBS. Among the composite-based resin cements, PA exhibited the most pronounced primer effect, with an initial SBS of approximately 20 MPa, whereas all specimens exhibited pretest debonding after thermal aging. This marked contrast suggests that the Visio.link/PA combination provided high initial bond strength but was susceptible to degradation during thermal cycling. The different responses of PA, RX, and GC may be related to differences in resin–matrix composition, viscosity, filler loading, polymerization chemistry, and compatibility with the polymerized primer layer. Nevertheless, because the chemical structure and degradation of the primer/cement interface were not directly investigated, the precise mechanism remains unclear. These findings further suggest that micromechanical retention generated by sandblasting alone is insufficient to achieve durable bonding when the resin cement has limited chemical interaction with the substrate. A similar concept has been reported for resin bonding to zirconia, where mechanical surface roughening alone does not necessarily provide durable adhesion without an appropriate chemical bonding strategy [37,38].

SB, an MMA-based resin cement, exhibited bonding behavior different from that of the composite-based resin cements. Under the initial condition, no significant difference in SBS was observed between the sandblasted and primer-treated PEEK groups. After aging, however, the sandblasted group showed significantly higher SBS than the primer-treated group. SB differs from the composite-based resin cements in that it contains 4-META together with an MMA-based resin matrix, which may contribute to its more favorable bonding behavior with sandblasted PEEK; however, the specific chemical contribution of 4-META was not directly evaluated in the present study. The MMA monomers in SB may have penetrated the surface micro-irregularities of sandblasted PEEK, thereby enhancing micromechanical interlocking. In addition, these monomers may have partially penetrated the superficial region of PEEK and contributed to the formation of an interpenetrating polymer network-like interface between PEEK and the MMA-based resin [39,40]. In contrast, in the primer-treated group, the primer layer may have covered the sandblasted surface and hindered direct penetration of the MMA monomers from SB into the surface irregularities. After aging, the primer layer itself may have functioned as a weak interfacial layer.

These findings indicate that the effect of the primer should not be interpreted simply as an improvement in bonding to PEEK. The adhesive primer may form an MMA-containing resin layer on the PEEK surface and enhance its compatibility with composite-based resin cements. Conversely, it may cover and smooth the roughened surface, thereby reducing micromechanical interlocking. Thus, primer application appears to exert two competing effects: improved compatibility with the resin cement and reduced micromechanical retention provided by the sandblasted PEEK surface. This may explain why the effectiveness of primer application depended strongly on the composition of the resin cement.

From a clinical perspective, the present findings provide useful information for optimizing bonding protocols for PEEK-based prosthetic materials under laboratory conditions. Alumina airborne-particle abrasion may contribute to improving the interaction between PEEK and resin cements; however, the selection of an adhesive primer should be considered according to the type of resin cement. An MMA-containing adhesive primer improved the initial bond strength of composite-based resin cements but did not enhance the bonding durability of the MMA-based resin cement evaluated in this study. Further clinical studies are required to determine whether these in vitro findings translate into improved long-term performance of PEEK crowns and frameworks.

Thermal cycling is a widely used in vitro aging method for evaluating the durability of dental adhesive interfaces; however, a standardized protocol has not yet been established, and various combinations of cycle numbers, temperatures, and dwell times have been reported in the literature [34]. In the present study, 20,000 thermal cycles were selected as an accelerated aging condition to evaluate the durability of the PEEK–resin cement interface. Nevertheless, thermal cycling represents only one aspect of the intraoral aging process. Clinical degradation may also be affected by other factors, such as occlusal loading, saliva exposure, pH changes, enzymatic degradation, and biofilm formation. Therefore, further studies incorporating additional aging factors are necessary to more comprehensively evaluate the long-term clinical durability of PEEK–resin cement bonding.

This study has several limitations. First, as an in vitro study, it could not fully reproduce the complex oral environment. Second, only one PEEK material and one MMA-containing adhesive primer were evaluated; therefore, the findings may not be generalizable to other PEEK formulations or primer systems. Third, surface topography was characterized using SEM, and the two-dimensional Ra parameter was measured with a stylus profilometer. Although this approach allowed for detection of the marked smoothing effect of primer application, three-dimensional parameters such as arithmetical mean height (Sa), maximum height (Sz), and developed interfacial area ratio (Sdr) would provide a more comprehensive characterization of the adhesive surface. In addition, untreated PEEK was not included because the present study focused on the additional effect of primer application after alumina airborne-particle abrasion. Finally, the thickness, continuity, and degree of polymerization of the primer layer, as well as the chemical and morphological characteristics of the PEEK/primer/resin cement interface, were not directly evaluated. Future studies should compare untreated, sandblasted, and primer-treated PEEK using three-dimensional surface analysis and should incorporate long-term water storage, thermomechanical aging, clinically relevant crown-retention testing, and detailed interfacial analyses.

## 5. Conclusions

Within the limitations of this in vitro study, an MMA-containing adhesive primer altered the surface characteristics of sandblasted PEEK and affected its bond strength to resin cements in a resin cement- and aging-dependent manner. Primer application improved the initial bond strength of composite-based resin cements but did not necessarily provide durable bonding after thermal aging. For the MMA-based resin cement SB, direct bonding to sandblasted PEEK was more favorable than bonding through a primer layer after aging. These findings suggest that the bonding protocol for PEEK should be selected according to the type and bonding mechanism of the resin cement rather than routinely including primer application.

## Figures and Tables

**Table 1:** Resin cements used in this study. The material compositions were obtained from information provided by the respective manufacturers.

Code	Type	Product name	Manufacturer	Composition
SB	MMA-based resin cement	Super-Bond EX	Sun Medical, Moriyama, Japan	MMA, PMMA, 4-META, TBB-O
RX	Composite-based resin cement	RelyX Universal Resin Cement	3M, Saint Paul, MN, USA	Dimethacrylate monomers, silica, glass powder, co-activator
GC	Composite-based resin cement	G-CEM LinkForce	GC, Tokyo, Japan	Bis-GMA, Bis-MEPP, UDMA, dimethacrylate monomers, barium glass, initiator
PA	Composite-based resin cement	Panavia V5	Kuraray Noritake Dental, Tokyo, Japan	Bis-GMA, TEGDMA, dimethacrylate monomers, silica, barium glass, aluminum oxide, initiator

MMA, methyl methacrylate; PMMA, poly(methyl methacrylate); 4-META, 4-methacryloxyethyl trimellitic anhydride; TBB-O, partially oxidized tri-n-butyl borane; Bis-GMA, bisphenol A diglycidyl methacrylate; Bis-MEPP, 2,2-bis(4-methacryloxypolyethoxyphenyl)propane; UDMA, urethane dimethacrylate; TEGDMA, triethylene glycol dimethacrylate.

**Table 2:** Failure modes after SBS testing and incidence of pretest debonding for sandblasted PEEK and primer-treated PEEK bonded with each resin cement. Pretest debonding was classified separately because the failure interface could not be examined.

Resin Cement	Surface Condition	Initial (A/M/C/P)	Aged (A/M/C/P)
SB	Sandblasted	8/2/0/0	10/0/0/0
SB	Primer-treated	10/0/0/0	10/0/0/0
RX	Sandblasted	10/0/0/0	0/0/0/10
RX	Primer-treated	10/0/0/0	10/0/0/0
GC	Sandblasted	10/0/0/0	0/0/0/10
GC	Primer-treated	10/0/0/0	10/0/0/0
PA	Sandblasted	10/0/0/0	0/0/0/10
PA	Primer-treated	10/0/0/0	0/0/0/10

Values indicate the number of specimens (n = 10). A, adhesive failure; M, mixed failure; C, cohesive failure; P, pretest debonding.

## Data Availability

The original contributions presented in this study are included in the article. Further inquiries can be directed to the corresponding author.
